# Polygenic prediction of breast cancer: comparison of genetic predictors and implications for risk stratification

**DOI:** 10.1186/s12885-019-5783-1

**Published:** 2019-06-10

**Authors:** Kristi Läll, Maarja Lepamets, Marili Palover, Tõnu Esko, Andres Metspalu, Neeme Tõnisson, Peeter Padrik, Reedik Mägi, Krista Fischer

**Affiliations:** 1 Estonian Genome Center, Institute of Genomics, University of Tartu, Riia 23b, 51010 Tartu, Estonia; 2 Broad Institute, Cambridge, MA USA; 3 Institute of Clinical Medicine, University of Tartu, Tartu, Estonia; 4 Cancer Center, Tartu University Hospital, Tartu, Estonia; 5 Institute of Mathematics and Statistics, University of Tartu, Tartu, Estonia; 6 Institute of Molecular and Cell Biology, University of Tartu, Tartu, Estonia

**Keywords:** Polygenic risk score, Genetic predisposition to disease, Breast cancer, Risk stratification, Personalized medicine

## Abstract

**Background:**

Published genetic risk scores for breast cancer (BC) so far have been based on a relatively small number of markers and are not necessarily using the full potential of large-scale Genome-Wide Association Studies. This study aimed to identify an efficient polygenic predictor for BC based on best available evidence and to assess its potential for personalized risk prediction and screening strategies.

**Methods:**

Four different genetic risk scores (two already published and two newly developed) and their combinations (metaGRS) were compared in the subsets of two population-based biobank cohorts: the UK Biobank (UKBB, 3157 BC cases, 43,827 controls) and Estonian Biobank (EstBB, 317 prevalent and 308 incident BC cases in 32,557 women). In addition, correlations between different genetic risk scores and their associations with BC risk factors were studied in both cohorts.

**Results:**

The metaGRS that combines two genetic risk scores (metaGRS_2_ - based on 75 and 898 Single Nucleotide Polymorphisms, respectively) had the strongest association with prevalent BC status in both cohorts. One standard deviation difference in the metaGRS_2_ corresponded to an Odds Ratio = 1.6 (95% CI 1.54 to 1.66, *p* = 9.7*10^− 135^) in the UK Biobank and accounting for family history marginally attenuated the effect (Odds Ratio = 1.58, 95% CI 1.53 to 1.64, *p* = 7.8*10^− 129^). In the EstBB cohort, the hazard ratio of incident BC for the women in the top 5% of the metaGRS_2_ compared to women in the lowest 50% was 4.2 (95% CI 2.8 to 6.2, *p* = 8.1*10^− 13^). The different GRSs were only moderately correlated with each other and were associated with different known predictors of BC. The classification of genetic risk for the same individual varied considerably depending on the chosen GRS.

**Conclusions:**

We have shown that metaGRS_2,_ that combined on the effects of more than 900 SNPs, provided best predictive ability for breast cancer in two different population-based cohorts. The strength of the effect of metaGRS_2_ indicates that the GRS could potentially be used to develop more efficient strategies for breast cancer screening for genotyped women.

**Electronic supplementary material:**

The online version of this article (10.1186/s12885-019-5783-1) contains supplementary material, which is available to authorized users.

## Background

Breast cancer (BC) is the most frequent cancer among women in the world, being also the second leading cause of cancer death in women in more developed regions after lung cancer [1]. As early diagnosis for BC could lead to successful treatment and good prognosis for recovery, it is important to develop efficient risk prediction algorithms that aid to identify high-risk individuals. Although many countries have implemented mammography screening programs, they are mostly applied to all women in certain age categories without any additional stratification by other risk factors. However, the benefits of such screening programs are often debated. Existing tools to assess BC risk [2–4] are often not systematically used in screening due to insufficient up-to-date risk factor’s information. Also, they only capture the heritable component either in the form of family history or using the information on rare genetic variants (BRCA1/2).

It has been estimated in twin studies that the heritability of breast cancer ranges from 20 to 30% [5]. However, only 5–10% of BC cases have a strong inherited component identified in a form of rare genetic variants [6], indicating that in addition there should be a considerable polygenic component in the disease liability. This is also supported by the results of large genome-wide association studies (GWAS) – more than 100 genomic loci have been identified as being associated with BC in Europeans [7].

Based on the GWAS results, several efficient polygenic risk scores (GRS) have been developed for common complex diseases that in many cases could be used to improve the existing risk prediction algorithms [8–11]. It is natural to expect that a similar GRS for BC may aid risk prediction in clinical practice.

So far, several studies have combined the SNPs with established genome-wide significance in a GRS for BC. Sieh *et al* [12] used 86 SNPs and Mavaddat *et al* [13] 77 SNPs to calculate a GRS, both showing a strong effect of the score in predicting future BC cases. Few studies have also demonstrated the incremental value of adding GRS to proposed BC prediction algorithms [14, 15]. Although several different GRSs have been proposed for BC risk prediction, no head-to-head comparison of the scores has been found in the literature. It has also not been assessed, whether the number of SNPs in the GRS could be increased. The latter was also problematic due to unavailability of summary statistics from large-scale GWASs.

In 2017, the large scale GWAS by Michailidou *et al* [7] released summary statistics for around 11.8 million genetic variants. Almost at the same time, UK Biobank released their GWAS results for BC for ~ 10.8 million SNPs. As evidence from studies on other common complex diseases have indicated that predictive ability of a GRS can be improved by adding the effects of a large number of independent SNPs in addition to the ones with established genome-wide significance, we intended to explore this approach using both summary files.

## Methods

### Study cohorts

In the present analysis, the data of 32,557 female participants of the Estonian Biobank (EstBB) [16] has been used, with 317 prevalent and 308 incident cases of BC. Incident disease data was obtained from linkages with the Estonian Health Insurance Fund, Estonian Causes of Death Registry and Estonian Cancer Registry (latest update in December 2015).

We have also analyzed the data of 46,984 women (incl 3157 BC cases) of European ancestry from the UK Biobank [17] who passed the main quality control and were not included in the UKBB breast cancer GWAS [18].

More details about cohorts can be found in the Additional file 2 and overview of the characteristics of the cohorts is given in the Additional file 1: Table S1.

### Statistical methods

#### General concept of genetic risk scores (GRS)

The general definition of a GRS was based on the assumption that the polygenic component of the trait (e.g. disease risk) can be approximated by a linear combination of *k* independent SNPs:

$$ {GRS}_i=\sum \limits_{j=1}^k{\beta}_j{X}_{ij} $$

where *β*_*j*_ is the weight of each SNP and *X*_*ij*_ represents the number of risk alleles for *j* − *th* SNP (*j* = 1, … , *k*) for the *i* − *th* individual, (*i* = 1,  … , *n*.). Typically the estimated (logistic) regression coefficients from a large-scale GWAS meta-analysis are used as weights *β*_*j*_.

Published versions of GRS can be divided to two main categories. We called a GRS *multigenic*, if the number of SNPs (*k*) is relatively small, containing only the SNPs with established genome-wide significance from a GWAS. A *polygenic* GRS contained a large number of SNPs (often *k* > 1000) and was either based on all available independent SNPs (with pairwise correlation not exceeding a pre-defined threshold) or the ones that satisfy some *p*-value threshold (often ≥0.05).

In the present paper, we computed two multigenic and two polygenic GRSs, whereas the polygenic GRSs were developed using the PRSice software [19].

#### Computation of multigenic and polygenic GRSs and analysis of their association with prevalent breast cancer

First we calculated two previously published multigenic GRSs for the EstBB data – both scores contained only those SNPs from the originally published versions that were available with acceptable imputation accuracy in the EstBB.

1. The score denoted by **GRS**_**70**_, based on Sieh et al [12](70 SNPs out of 86 were available).
2. The score **GRS**_**75,**_ based on the 75 SNPs of the 77-SNP score by Mavaddat *et al* [13]*.*

Next, polygenic GRSs were developed based on summary statistics of two different GWAS meta-analyses. First, two sets of independent SNPs were obtained so that: a) the SNPs with available summary statistics were genotyped or imputed with acceptable quality in the EstBB; b) the pairwise correlations between SNPs did not exceed a pre-specified threshold of r^2^ > 0.1 (more details on SNP selection provided in the Additional file 2). Subsequently, the selected SNPs were further filtered based on their *p*-value in the meta-analysis (using one of the pre-specified p-value thresholds). The corresponding effect estimates of the filtered subset were then used as weights to compose the GRSs. Altogether, we used 22 different p-value thresholds to compose 44 different versions of GRSs – 22 based on first meta-analysis and 22 based on the second one. To select the best predicting GRSs out of 44, age-adjusted logistic regression model comparing 317 prevalent BC cases and 2000 randomly chosen controls in the EstBB cohort was used and the scores with the smallest p-value for the GRS-phenotype association were selected (calculations about power to detect GRS-phenotype associations provided in Additional file 2). The resulting polygenic scores were:

3. The score **GRS**_**ONCO**_, based on the summary statistics of the Breast Cancer Association Consortium meta-analysis of BC with 122,977 cases and 105,974 controls [7].
4. The score **GRS**_**UK**_, based on the summary statistics of the GWAS conducted on the UK Biobank data (comparing 7480 BC cases and 329,679 controls including both men and women [18]). The reported linear regression coefficients were transformed into corresponding log odds ratios, following the rules described by Lloyd-Jones *et al* [20], before using them as weights in the GRS.
5. Thereafter, Pearson coefficients of correlation between all GRSs (GRS_70_, GRS_75_, GRS_ONCO,_ GRS_UK_) were calculated. Then GRSs were combined into three different versions of metaGRS, following the ideas by Inouye *et al* [21]: **metaGRS**_**4**_ as the weighted average of all four GRSs, **metaGRS**_**3**_ as the weighted average of three GRSs with the strongest association with incident BC and finally **metaGRS**_**2**_ based on top two predicting GRSs. To construct metaGRS, log (odds ratios) of GRSs from training set from logistic regression model were used as weights.

**Fig. 1:**
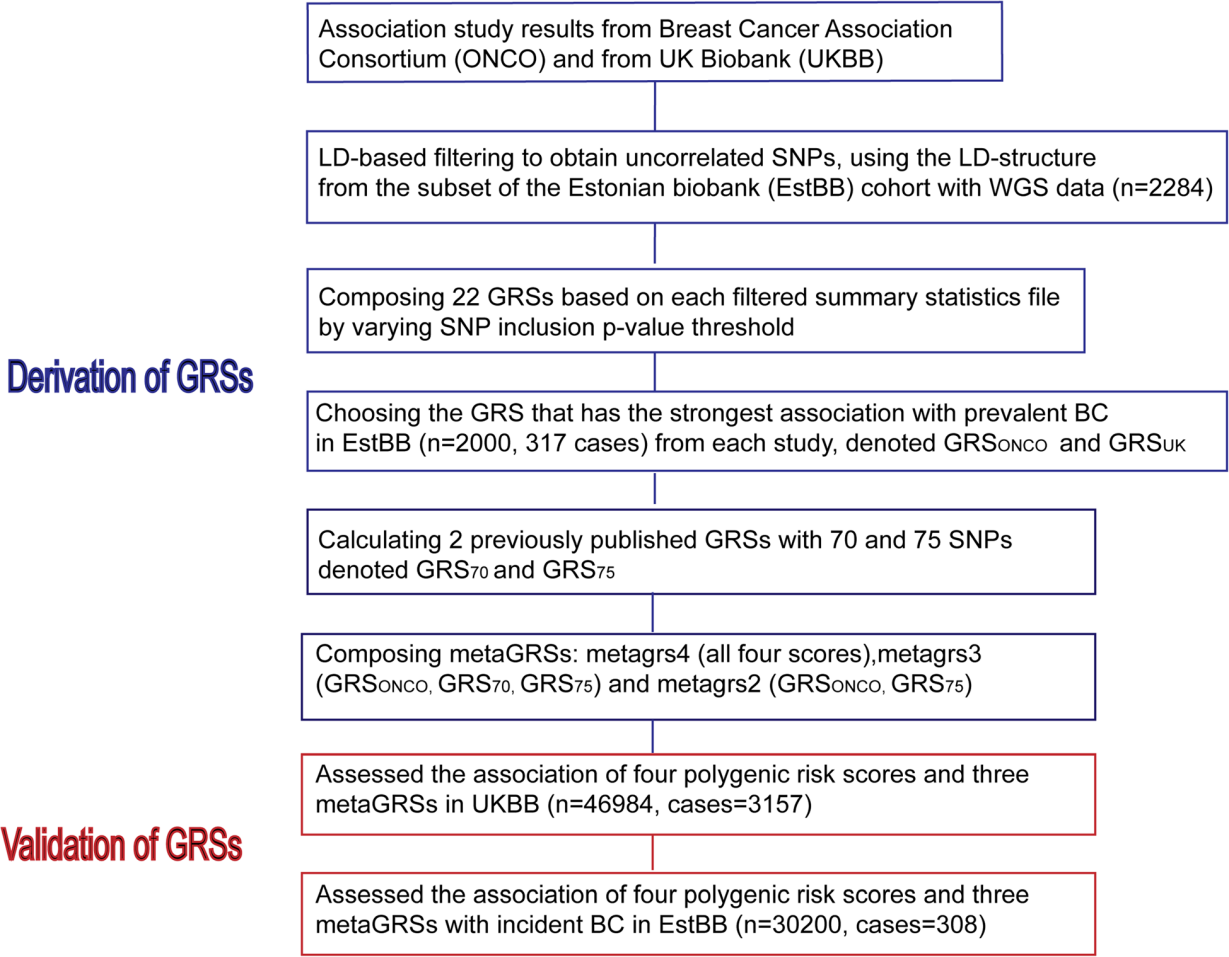
Workflow describing both derivation and validation of GRSs

Finally, the UK biobank data was used to further compare previously mentioned 7 GRSs and to address the attenuation of GRS’ effect while accounting for family history of BC and to study associations between BC risk factors and GRSs. While modelling in UK biobank, age at recruitment and 15 principal components were included in the model. The entire workflow was visualized in the Fig. 1.

#### Analysis of the GRS effects on incident BC

All 7 GRSs were evaluated in the analysis of incident BC in 30,240 women from the EstBB cohort who did not have an existing BC diagnosis at recruitment and were not included in the case-control set used to select the best polygenic GRSs. Cox proportional hazard models were used to estimate the crude and adjusted Hazard Ratios (HR) corresponding to one standard deviation (SD) of the GRS. To assess the incremental value of GRSs when added to other known risk factors, the models were additionally adjusted for the absolute risk estimates from the NCI Breast Cancer assessment tool [2, 22], based on age, race (for all participants, it was set to “White”, because only individuals with European decent were included), age at menarche and age at first live birth of the participant. Other possible risk factors such as number of biopsies were set as unknown. Harrell’s c-statistic to characterize the discriminative ability of each GRS and their incremental value compared to NCI’s Breast Cancer assessment tool absolute risk estimates alone were computed. Hazard ratios for GRS top quintile and top 5% percentile compared to average, median and low GRS categories were reported. Cumulative incidence estimates were computed with Aalen-Johansen estimator to account for competing risk. While comparing different GRS groups with each other, age was used as timescale to properly account for left-truncation in the data. While computing HR for continuous GRSs and comparing Harrell’s c-statistics alone and together with NCI estimates, follow-up time was used as timescale, as age is already included in NCI estimates.

Finally, associations between GRSs and variables related to female’s reproductive health and BC risk factors were explored using linear, logistic or Cox regression models depending on the type of dependent variable in both EstBB and UKBB cohorts (more details in the Additional file 2).

## Results

### GRSs association with prevalent breast cancer

Both GRS_70_ and GRS_75_ were significantly associated with prevalent BC status in the case-control subset of the EstBB cohort, with corresponding Odds Ratio (OR) estimates per one SD of the GRS being 1.27 (95% CI 1.13 to 1.45, *p* = 1.4*10^− 4^) and 1.38 (95% CI 1.22 to 1.57, *p* = 5.3*10^− 7^), respectively. Of all polygenic GRSs, the strongest association was observed for GRS_ONCO_ with *p*-value threshold *p* < 5* 10^− 4^ for SNP inclusion (898 SNPs). This resulted in OR = 1.44 (95% CI 1.27 to 1.64, p = 1*10^− 8^) per one SD of the GRS. The best version of GRS_UK_ included 137 SNPs that satisfied inclusion threshold p < 5*10^− 5^ and resulted in OR = 1.34 (95% CI 1.18 to 1.52, *p* = 5.5*10^− 6^). Similar effect sizes for all four GRSs were observed in the UKBB cohort (Additional file 1: Table S2). Detailed results on GRS-outcome associations in EstBB with different p-value thresholds for SNP inclusion can be seen in Additional file 2: Figure S1.

### Association of incident breast cancer and GRSs

Out of four studied GRSs, GRS_UK_ had the weakest and GRS_75_ the strongest association with incident BC (Table 1) in the EstBB, both in terms of the *p*-value as well as the Harrell’s c-statistic. All metaGRSs had stronger association with incident BC than original scores alone. However, when GRS_ONCO_ and GRS_75_ are already combined into metaGRS_2_, no additional gain was seen from adding GRS_UK_ and/or GRS_70_ to the score. Therefore, we chose metaGRS_2_ for further assessment of its properties. While a predictive model capturing the effect of the NCI risk estimates resulted in the Harrell’s c-statistic of 0.677, it was increased to 0.715 (by 3.8%) when also metaGRS_2_ was added to the model.

**Table 1:** Analysis results for incident breast cancer in EstBB using different GRSs and metaGRSs

Score	NCI	GRS_70_	GRS_75_	GRS_UK_	GRS_ONCO_	metaGRS_4_	metaGRS_3_	metaGRS_2_
HR^a^ per 1 SD with 95% CI	1.7 1.52–1.9	1.44 1.29–1.61	1.59 1.42–1.78	1.23 1.1–1.38	1.52 1.35–1.7	1.61 1.43–1.80	1.65 1.47–1.85	1.65 1.48–1.86
p-value	1.4*10^− 20^	3.2*10^− 10^	1.1*10^− 15^	4*10^− 4^	1.7*10^− 12^	4.4*10^− 16^	1.43*10^− 17^	7.6*10^−18^
Harrell’ s c –statistic	0.677	0.603	0.627	0.561	0.615	0.634	0.637	0.636
Harrell’ s c –statistic NCI + GRS	NA	0.701 (∆ = 0.024)	0.708 (∆ = 0.031)	0.684 (∆ = 0.007)	0.705 (∆ = 0.028)	0.715 (∆ = 0.038)	0.716 (∆ = 0.039)	0.715 (∆ = 0.038)

Legend: Harrell’s c-statistics for all versions of genetic risk scores and National Cancer Institute Breast Cancer Assessment Tool risk estimates (based on age, race, age at menarche and age at first live birth) were calculated. ∆-GRS added improvement in c-statistics compared to NCI alone. * Hazard ratio for developing breast cancer is given per 1 SD increase. CI = confidence intervals; GRS = genetic risk score; HR = Hazard ratio; NCI – National Cancer Institute Breast Cancer assessment tool estimates calculated with R package BCRA

No evidence of the interactions between any GRSs and NCI estimates were found (*p*-values > 0.16)

### The score metaGRS_2_ and its potential for personalized breast cancer risk prediction

**Fig. 2:**
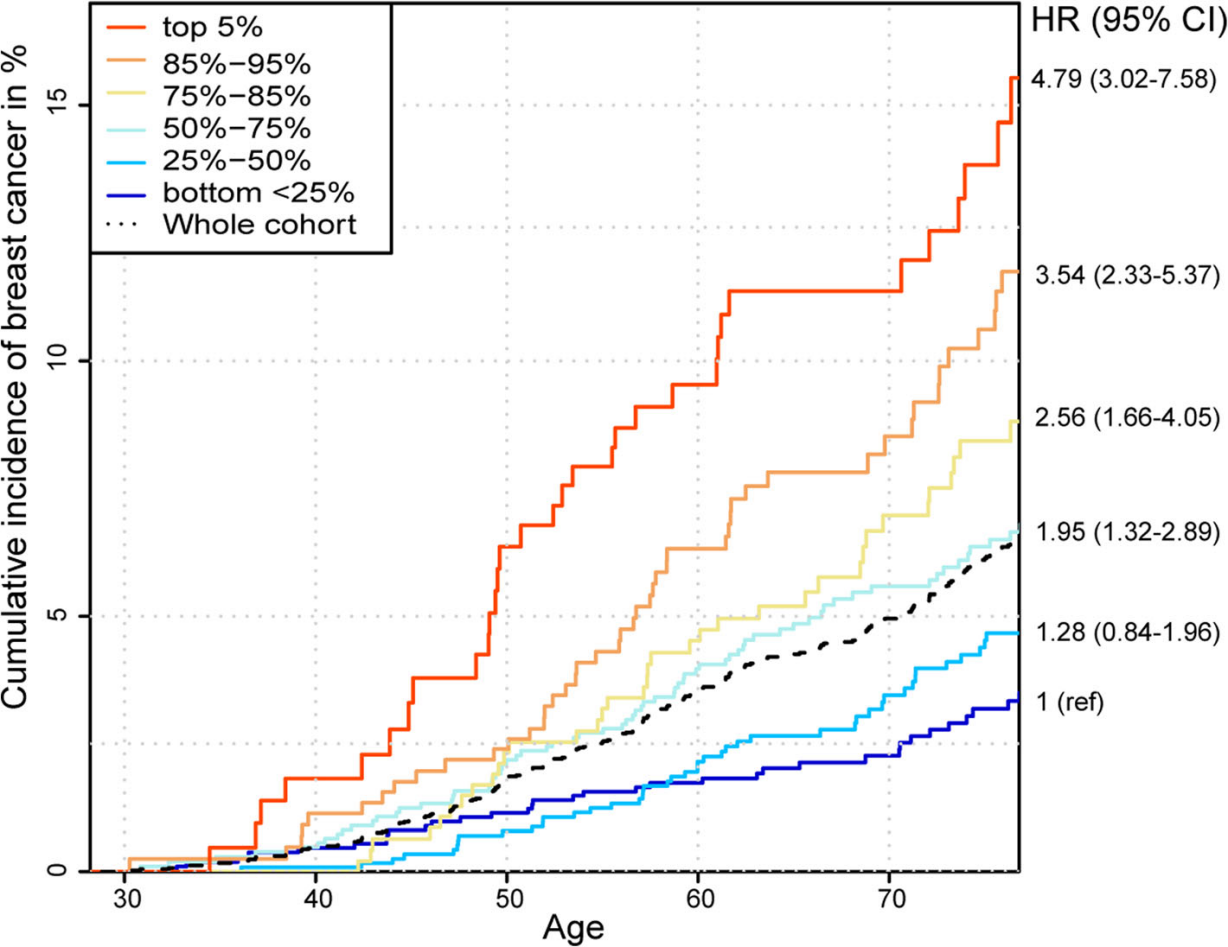
Cumulative incidence of BC in metaGRS_2_ categories among women within age 30–75 years. Legend: Cumulative incidence accounting for competing risks. Hazard ratios (HR) correspond to the comparison of several categories with the lowest quartile of metaGRS_2_

Women in the highest quartile of metaGRS_2_ distribution had 3.40 (95% CI 2.36 to 4.89) times higher hazard of developing BC than women in the lowest quartile. When the top quartile is further split into smaller percentiles (as seen on Fig. 2), a strong risk gradient was seen also within this quartile. Namely, women in the top 5% of the metaGRS_2_ distribution had a Hazard Ratio (HR) of 4.79 (95% CI 3.02 to 7.58) for incident BC compared to women in the lowest quartile, whereas HR = 4.20 (95% CI 2.84 to 6.23) for women in the top 5% compared to all women with metaGRS_2_ below the median. When the highest 5% percentile was compared with the rest of the cohort (women below the 95th percentile of metaGRS_2_), about three times higher hazard (HR = 2.73, 95% CI 1.92 to 3.90) was found. Compared to the women with metaGRS2 close to the median (belonging to the 40th to 60th percentile), the hazard of women in the top 5% of metaGRS2 was 2.7 (95% CI 1.77 to 4.18) times higher and the hazard of those with metaGRS2 below 40th percentile was almost 2 times lower (HR = 0.54, 95% CI 0.37 to 0.79) to develop BC.

As seen from Fig. 2, the cumulative BC incidence by the age of 70 was estimated to be 12% (95% CI 7.7 to 16.3%) for women in the top 5% percentile of metaGRS_2_, 8.3% (95% CI 5.6 to 11.0%) for those between 85 and 95% percentiles and 7.4% (95% CI 4.85 to 10.0%) for the women in 75–85% percentiles. Cumulative BC incidence in the third, second and first quartile of the metaGRS_2_ distribution was estimated to be 5.8% (95% CI 4.4 to 7.3%), 3.6% (95% CI 2.4 to 4.8%) and 2.4% (95% CI 1.4 to 3.3%), respectively. No significant difference in BC hazard was seen between the two lowest quartiles (*p* = 0.26), with both of them having considerably lower incidence level than the cohort average (overall cumulative BC incidence estimated as 5.1% by the age of 70, 95% CI 4.5 to 5.8%).

### Correlation of GRSs

**Fig. 3:**
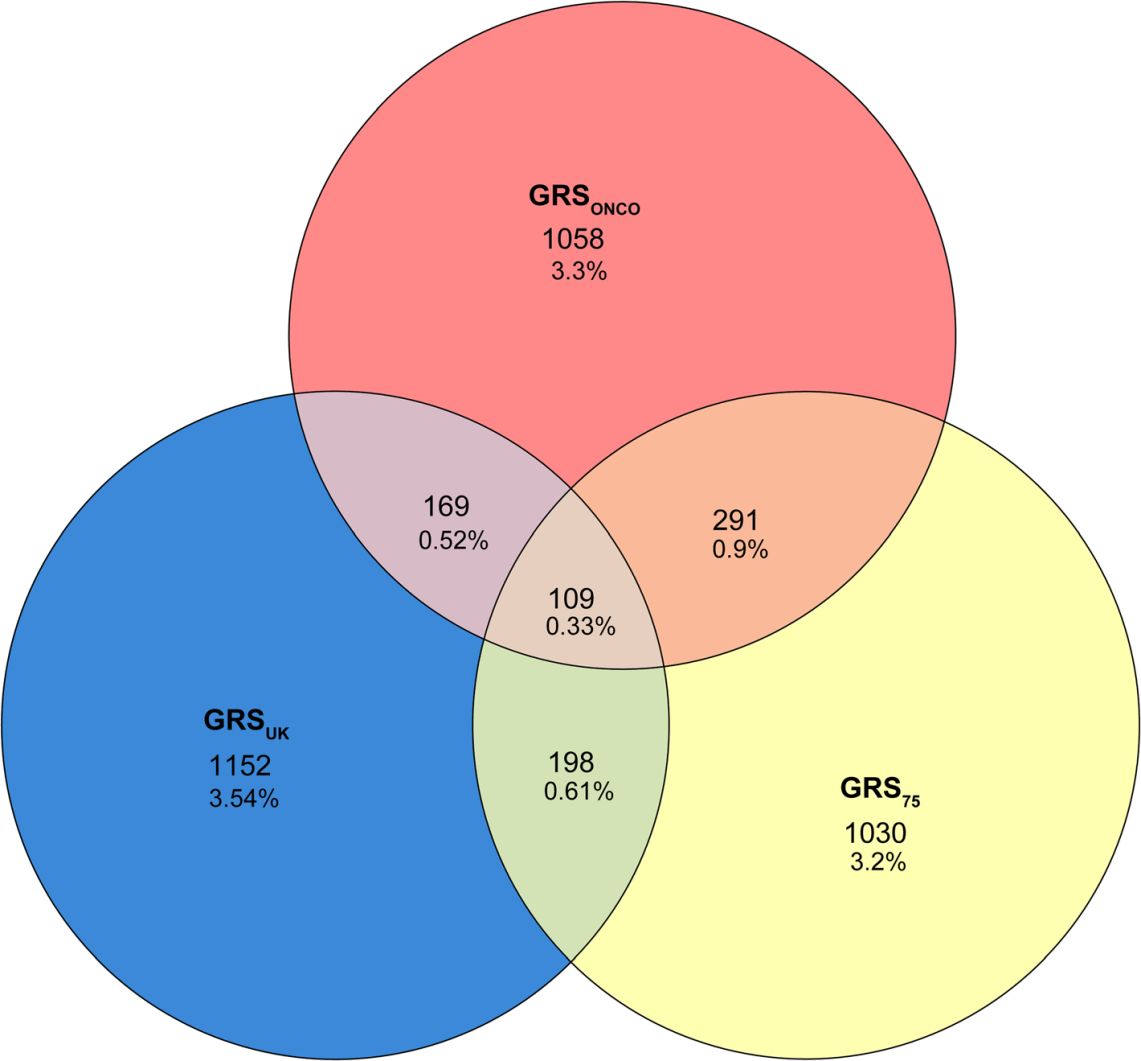
Division of Estonian Biobank women according to their genetic risk category. Legend: Women, who belong to top 5% at least with one out of the three genetic risk scores (GRSs: GRS_ONCO,_ GRS_UK,_ or GRS_75_), are represented on this graph. Number of women, who belong to top 5% only with one score, two scores or all three scores are given. Percentages are given per entire cohort

The correlations between seven scores varied between 0.3 to 1 (see Additional file 2: Figure S2). After dividing individuals into 2 categories (“non-high” – GRS < 95th percent and “high” – GRS in top 5%) based on three GRSs (GRS_UK,_ GRS_ONCO_ or GRS_75_), 87.7% (28547) of women were assigned to non-high category with all three scores. However, 12.4% (4010) of women belonged to high category with at least one GRS. 0.33% (109) of women belonged to top 5% with all three scores compared to ~ 10% (3240) of the women, who belonged into high category only with one score (Fig. 3).

### Associations of GRSs and other genetic and non-genetic predictors of breast cancer

Both family history as well as GRSs were strongly associated with BC status in UKBB, while the effects of GRSs were attenuated by less than 1% while adjusting for family history (Additional file 1: Table S2). The effect of family history was attenuated by 2.9–8.4%, depending on which GRS the model was adjusted for. For instance, the OR corresponding to the family history changed from 1.87 to 1.82 (corresponding to 2.9% change) while adjusting for the GRS_UK_ and to 1.71 (corresponding to 8.4% change) while adjusting for the metaGRS_2_. Known BC risk factors were only weakly associated with GRSs in both UKBB and EstBB cohorts (Additional file 1: Table S3-S4). BMI and waist circumference were negatively associated with GRS_UK_ in both EstBB and UKBB, the association in EstBB was stronger for women under 50 years of age. Smoking status was positively associated with all GRSs except GRS_UK_ only in EstBB data. Age at menopause was associated with some GRSs in both cohorts but the effects were in opposite direction. No GRS showed association with any other type of cancer or overall mortality.

## Discussion

We demonstrated that a metaGRS that combines a multigenic and a polygenic GRS for breast cancer - metaGRS_2_ - performed better than using either one of the previously published multigenic GRSs and also better than the best polygenic GRS alone. While in average about 5% of women in the EstBB cohort (as well as in the Estonian population) have been diagnosed with BC by the age of 70, women in the highest five percentiles of the metaGRS_2_ distribution reach the same cumulative risk level (5, 95% CI 2.1 to 7.8%) by the age of 49, thus more than 20 years earlier. It is also notable that women with metaGRS_2_ level below median reach such risk level (4.6, 95% CI 3.6 to 5.6%) only by age of 79, thus almost 10 years later. These findings suggest that the polygenic risk estimate based on metaGRS_2_ could be an efficient tool for risk stratification in clinical practice, for targeted screening and prevention purposes.

Given that the potential benefits of non-selective BC screening within certain age categories (compared to potential harm from over diagnosis) have been under serious discussion in the medical community [23], personalized approaches based on individual risk levels deserve further assessment. Ideally, those should integrate available information from clinical risk factors and also genetic information. The latter could include both moderate- and high-penetrance germline mutation testing, as well as polygenic risk scores. That approach is also supported by our findings, where considerable increase in c-statistics were observed while combining polygenic risk scores and NCI estimates together.

However, while incorporating a GRS in clinical BC prediction, one should keep in mind that a GRS represents a mixture of different pathways, but is still not likely to capture the heritable component completely. As our findings indicated that a GRS and family history have independent predictive effects on BC risk, accounting for individual’s genetic information and family history (indicating either the mother has suffered from breast cancer or not or the status is unknown) simultaneously seemed to result in the better risk estimation than using only one of these predictors alone. However, more research is needed to assess the usefulness of combining our proposed metaGRS_2_ with full pedigree-based family history data.

As depending on a GWAS that is used as a basis, different (and not necessarily highly correlated) GRSs can be produced, it can be expected that those GRSs might emphasize the effects of different biological pathways. This hypothesis seems plausible in the light of several associations found between different GRSs and BC risk factors. Expectedly, GRSs including only a small number of significant SNPs (like GRS_75_ and GRS_70_) were highly correlated and if we could have included all original 86 SNPs instead of 70, correlation between GRS_86_ and GRS_75_ would have likely remained similar or decreased a little, as excluded SNPs from the original 86 SNPs were rather rare.

The fact that a metaGRS performed better than alternatives, suggests that even though the multigenic GRS_75_ including only genome-wide significant SNPs was already a good predictor for BC, other SNPs included in the polygenic GRS_ONCO_ - but not in the GRS_75_ - have some additional predictive power. Most likely, not all SNPs included in the GRS_ONCO_ are truly associated with BC, however, as they have some predictive power, possibly also through being associated with some of the risk factors of BC, one should not completely ignore them while building an optimal GRS.

It remains an open question whether it is always the best practice to use metaGRS instead of several different genetic risk scores – if one can pinpoint biological mechanisms behind different scores, more optimal preventive strategies could be chosen. Still, until we are unable to convincingly link different GRSs with specific preventive measures, targeted prevention should be based on a GRS with the best possible overall predictive ability, such as the metaGRS_2_ proposed here.

One should also keep in mind that besides GRS there are genetic mutations such as BRCA1/2 known to be associated with very high familiar BC risk. Therefore, in practice, any genomic risk stratification procedure should also include search for high- and moderate-risk genetic variants, if possible. In the high-risk mutation carriers, the clinical management could be based on the specific genetic (mendelian) variants, or if deemed useful in the future, a combination of mendelian variants and GRS levels, but it definitely needs further studies.

## Conclusions

In summary, our results showed that an efficient polygenic risk estimate enables to identify strata with more than four-fold differences in BC incidence. This definitely calls for the development of personalized screening and prevention strategies that incorporate the GRS information, having the potential to considerably increase the benefits of nation-wide screening programs and reduce the existing controversies on their efficacy. However, one should be aware of the fact that a GRS is still a proxy of a true genetic risk and it is not uniquely defined – as more research accumulates, more efficient polygenic predictors could be developed that may re-categorize some previously stratified individuals into high or low risk groups. In addition, a GRS should ideally be combined with information on other genetic and non-genetic risk factors for best possible accuracy in risk assessment.

## Additional files

Additional file 1: **Table S1.** Cohort characteristics of UK Biobank and Estonian Biobank. **Table S2.** Associations of breast cancer and standardized GRSs in the UK Biobank (with and without adjustment of family history) and in Estonian Biobank without family history. **Table S3.** Associations between GRSs and risk factors of breast cancer in Estonian Biobank. **Table S4.** Associations between GRSs and risk factors of breast cancer in UK Biobank. (XLSX 30 kb)

Additional file 2: **Figure S1.** Associations of GRSs with prevalent breast cancer in EstBB data. **Figure S2.** Correlations between different genetic risk scores (GRSs). **Figure S3.** Power to detect an association between GRS and breast cancer status given the sample size of the case-control and prevalence of the disease. (DOCX 128 kb)

## Data Availability

We do not have ethical approval to share individual level genotype and phenotype data for Estonian Biobank. The data from UK Biobank were used under license for the current study, and so are not publicly available. Researchers interested in Estonian Biobank can request the access here: https://www.geenivaramu.ee/en/access-biobank and access to UK Biobank can be requested here http://www.ukbiobank.ac.uk/resources/.

## Abbreviations

BC: Breast Cancer
CI: Confidence Intervals
EstBB: Estonian Biobank
GRS: Genetic Risk Score
GWAS: Genome-Wide Association Study
HR: Hazard Ratio
metaGRS: combination of several genetic risk scores number in subscript indicates the number of original GRSs included
NCI: National Cancer Institute Breast Cancer
OR: Odds Ratio
SD: Standard Deviation
SNP: Single Nucleotide Polymorphism
UKBB: UK Biobank
