# Supplementary material for: Polygenic prediction of breast cancer: comparison of genetic predictors and implications for risk stratification
Source: BMC Cancer. 2019 Jun 10;19:557. doi: 10.1186/s12885-019-5783-1 (PMC6558751; doi:10.1186/s12885-019-5783-1)
Supplement: Supplementary file 2 — Figure S1. Associations of GRSs with prevalent breast cancer in EstBB data. Figure S2. Correlations between different genetic risk scores (GRSs). Figure S3. Power to detect an association between GRS and breast cancer status given the sample size of the case-control and prevalence of the disease. (DOCX 128 kb) [file 12885_2019_5783_MOESM2_ESM.docx]

# Supplementary Material and Methods

## Genotyping, imputation and quality control

EstBB: Genotyping was done in the Core Genotyping Lab in University of Tartu and variants were called using Illumina GenomeStudio and additionally by zCall for Illumina GSA and CE arrays. Before imputation all genotyped datasets were quality controlled. Samples with call-rate <95%, had gender mismatch between genotype and questionnaire or genotype heterozygosity more extreme than ±mean×3sd were excluded. Genetic variants with call-rate<95%, HWE p-value <1e-6, MAF<1%, and palindromic variants were excluded. The genotypes of all samples were imputed using the Estonian population based whole genome sequencing dataset as the reference panel^4^. Imputation was done using two-stage approach: EAGLE v. 2.3^5^ for phasing and BEAGLE v. 21Jan17^6^ for imputation. Estimated genotypes were generated for approximately 38 million SNPs.

UK Biobank: As part of the quality control we excluded all males and the females marked with poor heterozygosity or missingness, having excess (>10) of relatives or putative sex chromosome aneuploidy. We only used samples that were included in autosome phasing. Further QC was done by excluding samples with mismatching genetic and self-reported sex or ethnicity. Finally, we extracted the set of unrelated women while trying to keep as many breast cancer cases as possible. Cases were defined as the union set of women with ICD-10 code C50 and self-reported breast cancer (code: 1002). Furthermore, we extracted data about age at menarche and natural menopause, smoking status, use of hormonal replacement therapy, BMI, waist circumference, knowledge of participant’s mother ever having suffered from breast cancer (later referred to as family history, unknown for about 10% of participants in the entire UKBB dataset and 7.7% in our subset of UKBB, coded as 0-no, 1-yes, 2-unknown) and diagnosis status for endometriosis, polycystic ovarian syndrome and any cancer. For anthropometric measurements we use the data collected from the initial assessment (at recruitment). Only individuals, who were not included in the UKBB GWAS for BC, were used during the analysis. UKBB participants were genotyped on two different arrays: UK BiLEVE array (3926) and UK Biobank array (43058). The genetic data had been imputed using the Haplotype Reference Consortium (HRC) panel as reference.

## GWAS data management

For SNP and weight selection for developing GRSs, summary statistics from two GWAS analyses were used: the study conducted on the UK Biobank data (comparing 7,480 breast cancer cases and 329,679 controls including both men and women)^3^ and large meta-analysis of breast cancer (including Breast Cancer Association Consortium and ICOGS study) with 122,977 cases and 105,974 controls^7,8^.

As a first step, summary statistics were filtered for minor allele frequency (a threshold of 2% was imposed). For our study, independent set of SNPs were obtained using clumping procedure implemented in PLINK-1.9^9^ with following parameters (--clump-p1=1, --clump-r2=0.1, --clump-kb=500). Estonian whole genome sequencing dataset (n=2,284) was used as a reference panel to obtain LD structure between the SNPs. After clumping and data quality control, 231,498 SNPs from UK biobank GWAS and 143,358 SNPs on Breast Cancer Association Consortium study remained, respectively. For any version of GRS calculation, only bi-allelic SNPs with both MAF exceeding 1% and accurate imputation (squared correlation between known genotypes from the WGS data and imputed allele dosages (R^2^) > 0.8) in EstBB data were included. The criteria for accurate imputation was taken from^10^. Different versions of GRS (with varying number of SNPs included) were calculated using the PRSICE.2.0 software^11^. Only SNPs (k=1107) included in the best predicting GRSs were retrieved from UK biobank dataset. Out of these, 32 were excluded from Breast Cancer Association Consortium based GRS due to imputation quality.

## Statistical analysis

Power analysis of GRS development

Our case-control dataset with 317 cases and 2000 controls was used to compare the 44 versions of GRSs (22 based on the UKBB GWAS and 22 based on the BCAC analysis) and to choose the best predicting version. We performed a power analysis to investigate the power to detect GRS-phenotype associations with given sample size and prevalence: the sample size was set to 2317 and prevalence in this sample was set to 13.7% (corresponding to 317 cases). Using the significance level of 0.05, we calculated the power to detect an association between a GRS and breast cancer status by logistic regression analysis, by varying the true effect size of a GRS. Results can be seen on the Figure S3. When the OR of a standardized GRS is larger than 1.2, the power to detect the association between a GRS and BC status would exceed 80%. As the OR-s corresponding to each of the four GRSs (GRS_75_, GRS_70_, GRS_UK_ and GRS_ONCO_) were estimated to be larger than 1.25, we believe that we have a reasonable sized training set to compare different versions of GRSs with at least moderate effect size. The calculations were done using the R package “WebPower”.

Association analysis of BC risk factors and different GRS

In EstBB cohort, associations between incident cases of endometriosis, polycystic ovarian syndrome, ovarian cancer or any cancer were investigated with cox regression model using age at recruitment as time scale. Linear regression models were fitted for NCI score, age at menarche and age at menopause. Logistic regression models adjusted for age at recruitment were fitted for the current smoking status and the use of hormonal contraceptives.

In UK biobank, associations for all binary variables were modelled with logistic regression. Models were adjusted for first 15 principal components and age at recruitment.

## References

1. Leitsalu, L. *et al.* Cohort Profile: Estonian Biobank of the Estonian Genome Center, University of Tartu. *Int. J. Epidemiol.* dyt268- (2014). doi:10.1093/ije/dyt268

2. Sudlow, C. *et al.* UK biobank: an open access resource for identifying the causes of a wide range of complex diseases of middle and old age. *PLoS Med.* **12,** e1001779 (2015).

3. Ben Neale Lab. Rapid GWAS of thousands of phenotypes for 337,000 samples in the UK Biobank — Neale lab. (2017). Available at: http://www.nealelab.is/blog/2017/7/19/rapid-gwas-of-thousands-of-phenotypes-for-337000-samples-in-the-uk-biobank. (Accessed: 2nd May 2018)

4. Mitt, M. *et al.* Improved imputation accuracy of rare and low-frequency variants using population-specific high-coverage WGS-based imputation reference panel. *Eur. J. Hum. Genet.* **25,** 869–876 (2017).

5. Loh, P.-R., Palamara, P. F. & Price, A. L. Fast and accurate long-range phasing in a UK Biobank cohort. *Nat. Genet.* **48,** 811–816 (2016).

6. Browning, B. L. & Browning, S. R. A Unified Approach to Genotype Imputation and Haplotype-Phase Inference for Large Data Sets of Trios and Unrelated Individuals. *Am. J. Hum. Genet.* **84,** 210–223 (2009).

7. Michailidou, K. *et al.* Association analysis identifies 65 new breast cancer risk loci. *Nature* **551,** 92–94 (2017).

8. BCAC - The Breast Cancer Association Consortium. OncoArray and Combined Summary results. (2017). Available at: http://bcac.ccge.medschl.cam.ac.uk/bcacdata/oncoarray/. (Accessed: 4th May 2018)

9. Purcell, S. *et al.* PLINK: a tool set for whole-genome association and population-based linkage analyses. *Am. J. Hum. Genet.* **81,** 559–75 (2007).

10. Liu, Q. *et al.* Systematic assessment of imputation performance using the 1000 Genomes reference panels. *Brief. Bioinform.* **16,** 549–62 (2015).

11. Euesden, J., Lewis, C. M. & O’Reilly, P. F. PRSice: Polygenic Risk Score software. *Bioinformatics* **31,** 1466–8 (2015).

# Supplementary Figures
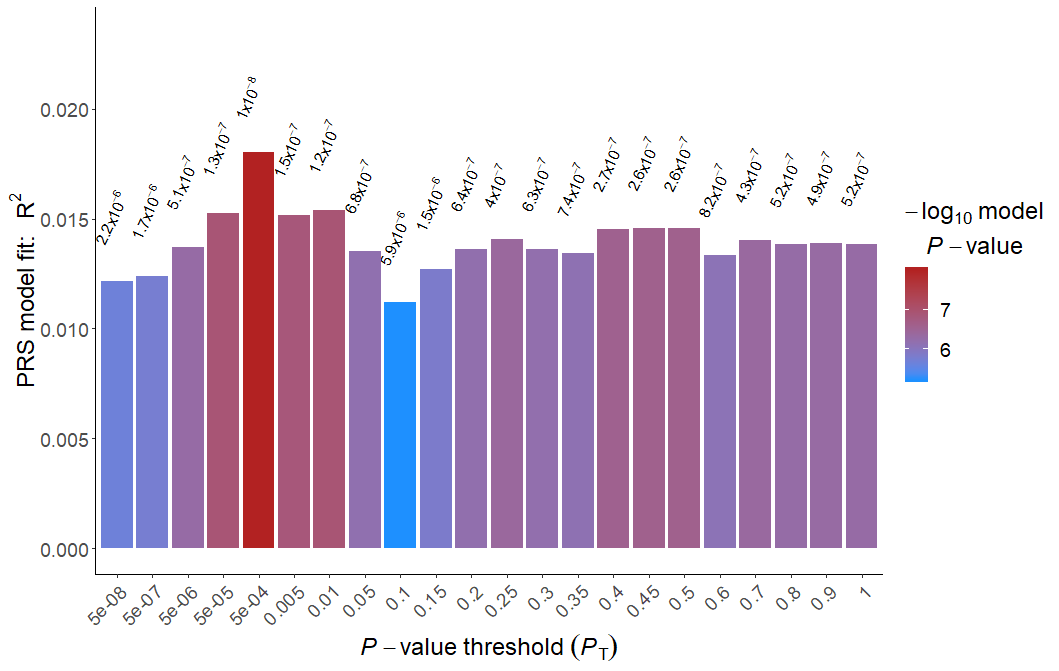


A)

B)

Figure S1. Association of GRSs with prevalent breast cancer in EstBB data. The A) figure shows the GRSs based on Breast Cancer Association Consortium (ONCO) GWAS data and the B) figure shows GRSs based on UK Biobank GWAS, respectively. The highest pseudo-R2 is achieved with inclusion p-value threshold $\boldsymbol{p<} \boldsymbol{5*10}^{\boldsymbol{-4}}$ in ONCO and with $\boldsymbol{p<} \boldsymbol{5*10}^{\boldsymbol{-5}}$ in UK biobank based GWAS.


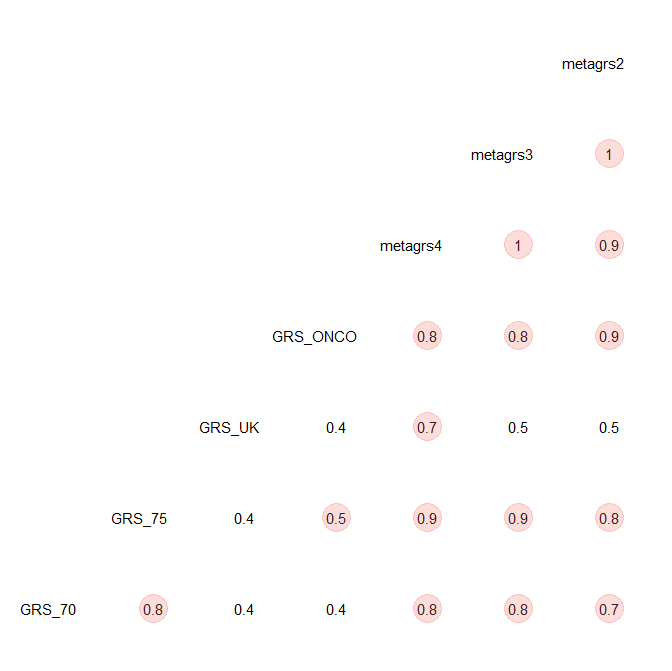


Figure S2. Correlations between different genetic risk scores (GRSs).


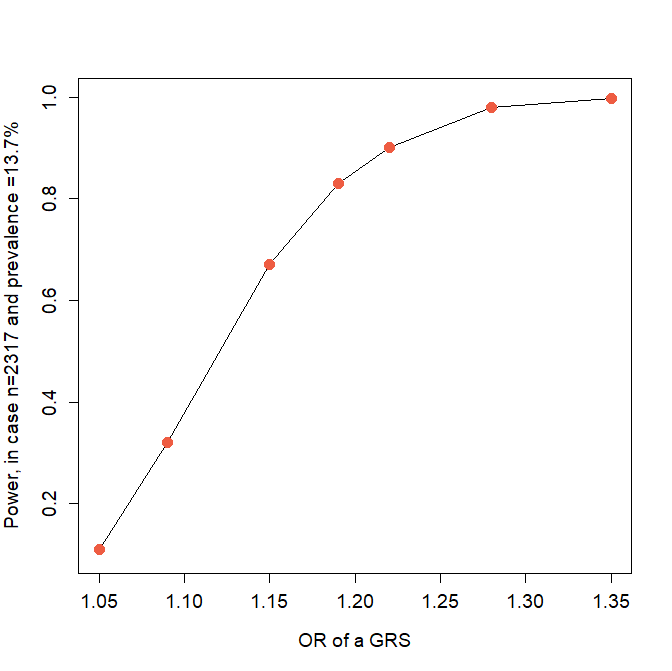


Figure S3. Power to detect an association between GRS and breast cancer status given the sample size of the case-control and prevalence of the disease.
